# Multidrug-Resistant and Carbapenemase-Producing *Enterobacteriaceae* in Addis Ababa, Ethiopia

**DOI:** 10.1155/2021/9999638

**Published:** 2021-06-11

**Authors:** Saba Gebremichael Tekele, Dejenie Shiferaw Teklu, Melese Hailu Legese, Daniel Gebretsadik Weldehana, Melaku Ashagrie Belete, Kassu Desta Tullu, Samuel Kinde Birru

**Affiliations:** ^1^Department of Medical Laboratory Sciences, College of Medicine and Health Sciences, Wollo University, Dessie, Ethiopia; ^2^Department of Bacteriology and Mycology, Ethiopian Public Health Institute, Addis Ababa, Ethiopia; ^3^Department of Medical Laboratory Sciences, College of Health Sciences, Addis Ababa University, Addis Ababa, Ethiopia

## Abstract

**Background:**

The emergence and spread of multi-drug resistant (MDR) bacteria have become a public health problem in recent years. For the last many years, carbapenem antibiotics have been used successfully to treat infections caused by MDR *Enterobacteriaceae*. However, recently, *Enterobacteriaceae* producing carbapenemases have emerged, which confer broad resistance to most *β*-lactam antibiotics including carbapenems. Therefore, this study is aimed at determining the magnitude of MDR and carbapenemase-producing *Enterobacteriaceae* (CPE) isolated from various clinical specimens in Addis Ababa, Ethiopia.

**Methods:**

A cross-sectional study was conducted from January to April 2018. A total of 312 *Enterobacteriaceae* isolates were identified from various clinical specimens. The Phoenix automated system (BD Phoenix100) was used for bacterial identification and antimicrobial susceptibility testing. Potential carbapenemase producers were confirmed by the modified carbapenem inactivation test, and KPC, MBL, and OXA-48 were phenotypically characterized by the disk diffusion method. The data obtained were entered and analyzed using SPSS version 20 software. Descriptive statistics, chi square, bivariate and multivariable logistic regression analyses were performed. *P* value ≤ 0.05 with corresponding 95% confidence interval was considered for statistical significance.

**Results:**

A total of 312 *Enterobacteriaceae* were recovered. Of these isolates, 68.6% were MDR and 2.6% were CPE with different classes including OXA-48 1.6% (5/312), MBL 0.6% (2/312), and KPC and OXA-48 0.3% (1/312). The predominant bacterial isolates were *E. coli* 72.4% (226/312) followed by *K. pneumoniae* 13.8% (43/312). The antibiotic resistance rates of CPE isolates were significantly higher than other MDRE including ampicillin (100% versus 77.6%), cefoxitin (75% versus 20.6%), and piperacillin/tazobactam (50% versus 13.1%).

**Conclusion:**

In this study, a relatively higher prevalence of MDR was observed, and the highest resistance was recorded against ampicillin, amoxicillin with clavulanic acid, and sulfamethoxazole-trimethoprim. Detection of CPE is important for implementing appropriate antimicrobial therapy and in controlling the spread of the infection. Furthermore, continuous screening and investigations, including genotypic characterization of CPE, are required for the prevention and control of the spread of antimicrobial-resistant pathogens.

## 1. Background

The emergence and spread of multi-drug resistant (MDR) bacteria have become a public health problem in recent years [1]. Of particular concern are infections caused by resistant *Enterobacteriaceae*, which are common pathogens causing different types of community- and hospital-acquired infections, and antimicrobial resistance in these bacteria has significant impacts on patient outcomes [2].

For the last many years, carbapenem antibiotics have been used successfully to treat infections caused by multi-drug resistant *Enterobacteriaceae*, including those producing extended spectrum *β*-lactamases (ESBL). However, recently, *Enterobacteriaceae* producing carbapenemases have emerged, which confer broad resistance to most *β*-lactam antibiotics including “last-line” carbapenems [1].

Even though there are several mechanisms for the resistance of carbapenem such as a decrease in permeability of bacterial outer membrane with overexpression of AmpC/ESBL or efflux pump, the main mechanism of carbapenem resistance is the production of carbapenemase [3]. The most common carbapenemases include veronica integron metallo-*β*-lactamase types (VIM), imipenemase (IMP), *K. pneumoniae* carbapenemase (KPC), oxacillinase-48 (OXA-48), and New Delhi metallo-*β*-lactamase-1 (NDM-1) [4]. These carbapenemase genotypes differed geographically. For instance, OXA-48 enzymes particularly recovered within the Mediterranean area, including Northern Africa and Middle East [5].

According to the World Health Organization (WHO) recent report, CPE has listed among the antibiotic-resistant bacteria with a level one (critical) priority for research and development of new antibiotics [6].

The prevalence of carbapenem-resistant *Enterobacteriaceae* (CRE) infections has increased over the last decade, especially in healthcare settings, and the Centers for Disease Control and Prevention (CDC) estimates that more than 9000 healthcare-associated infections are caused by CRE each year in the United States [7, 8]. Moreover, CRE can cause a number of serious infections such as intra-abdominal infections, pneumonia, urinary tract infections, and device-associated infections [9–11]. The mortality rates are also high and range from 18% to 48% depending on therapy [12].

Currently in Ethiopia, different studies showed that there is increasing use of carbapenem in health facilities or physicians use carbapenem for empirical treatment. Due to this, treatment options for serious CRE infections remain limited. Optimization of dosing of currently available agents and combination therapy may be the most appropriate treatment strategies at this time. However, continued research is desperately needed, in particular randomized controlled trials, to determine the most appropriate treatment for serious CRE infections. Therefore, this study is aimed at determining the magnitude of MDR and carbapenemase-producing *Enterobacteriaceae* isolated from various clinical specimens in Addis Ababa, Ethiopia.

## 2. Methods

A cross-sectional study was conducted at International Clinical Laboratories (ICL) in Addis Ababa, Ethiopia, from January 1 to April 30, 2018. A total of 312 *Enterobacteriaceae* isolates were recovered from different clinical specimens and included using a convenient sampling technique for carbapenemase characterization and antimicrobial susceptibility testing. A predeveloped worksheet was used to collect information related to sociodemographic characteristics of the patients. The identified *Enterobacteriaceae* isolates, the antibiotic susceptibility pattern of the isolate, and classes of carbapenemases were also recorded using a separate data collection sheet.

### 2.1. Cultivation and Identification of Isolates

Different clinical specimens were inoculated onto appropriate culture media (sheep blood agar, XLD agar, and MacConkey agar plates (Oxoid Ltd, UK)) and incubated overnight under the aerobic condition at 37°C for 18-24 hours. Identification of *Enterobacteriaceae* was done using colony characteristics, Gram staining reaction, and ability to ferment lactose. In addition, the Phoenix system (BD Diagnostic Systems, Oxford, UK) was used for the identification of the bacteria to species level. The combination panel includes identification (ID) side with dried substrates for bacterial identification, and the instrument tests panels every 20 minutes. After three hours, the identified bacteria were displayed on the screen of the Phoenix system [13].

### 2.2. Antimicrobial Susceptibility Testing

Antimicrobial susceptibility testing was performed using the Phoenix AST panel (AST-N94). The following antimicrobials were included: ceftazidime, cefuroxime, ciprofloxacin, ceftriaxone, cefepime, amoxicillin with clavulanic acid, amikacin, aztreonam, ertapenem, cefoxitin, gentamicin, imipenem, meropenem, ampicillin, sulfamethoxazole-trimethoprim, and piperacillin/tazobactam [13]. Results were interpreted according to CLSI recommendations [14]. Multi-drug resistant isolates were determined using the definition of Magiorakos et al. [15].

### 2.3. Carbapenemase Detection

Potential carbapenemase-producing isolates, which showed resistance to at least one of the tested carbapenems (ertapenem, imipenem, and meropenem) were selected when MIC ≥ 2 *μ*g/ml for imipenem, ≥1 *μ*g/ml for ertapenem, and/or ≥2 *μ*g/ml for meropenem [13].

Suspected carbapenemase-producing isolates were confirmed by the modified carbapenem inactivation methods (mCIM) which are recommended by CLSI [14]. After preparation of bacterial suspension of tested isolates in 2 ml Trypticase soya broth, meropenem disk (10 *μ*g) was added and incubated at 35°C ± 2°C in ambient air for 4 hours ± 15 minutes. When the time was completed, meropenem disk (10 *μ*g) was placed on a Muller Hinton agar plate inoculated with *E. coli* ATCC 25922 suspension of 0.5-McFarland turbidity standards and incubated overnight (18–24 hrs.) at 37°C. Bacterial isolates having a zone of inhibition 6–15 mm or presence of pinpoint colonies within a 16–18 mm zone and no inhibition of the meropenem-susceptible *E. coli* ATCC 25922 were confirmed as carbapenemase-producing isolates.

### 2.4. Phenotypic Characterization of Carbapenemases

Carbapenemases were characterized phenotypically by a disk diffusion method using Neo-Sensitabs disks. The organism to be tested was spread onto a Mueller Hinton agar plate using similar procedures as for drug susceptibility testing. A meropenem (10 *μ*g) disk alone and in combination with inhibitors of different beta-lactamases such as phenylboronic acid (KPC and AmpC inhibitor), dipicolinic acid (MBL inhibitor), cloxacillin (AmpC inhibitor), and temocillin disks (30 *μ*g) (Rosco, Taastrupgaardsvej, Denmark) was used; then, the organism was incubated at 37°C for 24 hours. An increase in the inhibition zone diameter for a combination disk versus meropenem disk alone was interpreted as a carbapenemase producer [16].

### 2.5. Quality Assurance

To maintain the quality of the work from isolate collection up to final bacterium identification and data management, the standard operating procedure of isolate collection and laboratory analysis was strictly followed. The expiry date of the media, reagents, and antibiotic disks was checked before use. The prepared culture media was checked for sterility. Abilities of the prepared media supporting the growth of organisms were checked by inoculating ATCC control strain including *S. aureus* (ATCC 25923), *E. coli* (ATCC 25922), *E. faecalis* (ATCC 29212), and *P. aeruginosa* (ATCC 27853) [14]. Quality control testing for the Phoenix machine was done for each lot of panels [13]. For carbapenemase detection, BAA1705 control strains as positive control and BAA1706 as negative control were used [16].

### 2.6. Data Analysis and Interpretation

Data was collected using a worksheet and analysed using SPSS version 20 software. Descriptive statistics, chi square, and bivariate and multivariable logistic regression analyses were performed. *P* value ≤ 0.05 with corresponding 95% confidence interval was considered for statistical significance.

Frequency as well as percentages of MDR and carbapenemase-producing *Enterobacteriaceae* was calculated. Moreover, data was presented using tables and graphs.

## 3. Results

### 3.1. Magnitude of *Enterobacteriaceae*

A total of 312 *Enterobacteriaceae* isolates were isolated from various clinical specimens sent to the microbiology laboratory. Of these isolates, 58.0% (*n* = 181/312) were from females, while 42.0% (*n* = 131/312) were from males with a mean (standard deviation) age of 44.2 (21.8) years, and 67.3% (*n* = 210/312) of them were inpatients. About 77.2% (241/312) of *Enterobacteriaceae* were isolated from urine, and 16.7% (52/312) were from pus. Among *Enterobacteriaceae* isolates, *E. coli* was the dominant isolate accounting for 72.4% (226/312), and *K. pneumoniae* was the second predominant species representing 13.8% (43/312) of the total isolates. About 84.9% (192/226) of *E. coli* was isolated from urine, and 11.1% (25/226) were from pus specimens (Table 1).

### 3.2. Multi-drug Resistance Patterns of *Enterobacteriaceae*

Out of the total 312 *Enterobacteriaceae* isolate enrolled in this study, 68.6% (214/312) were MDR. The principal MDR isolates were *Enterobacter* spp. (90.9), *Citrobacter* spp. (81.8), *K. pneumoniae* (79.1), and *E. coli* (68.8) (Table 2).

### 3.3. Carbapenemase-Producing *Enterobacteriaceae*

From the total isolates, 17 isolates were potential carbapenemase producers. Of these isolates, 23.5% (8/17) was confirmed as a carbapenemase producer by mCIM. The overall magnitude of carbapenemase-producing *Enterobacteriaceae* was 2.6% (8/312) which includes *K. pneumoniae* 1.3% (4/312), *E. coli* 0.9% (3/312), and *Enterobacter* spp. 0.3% (1/312).

Similarly, those confirmed carbapenemase-producing *Enterobacteriaceae* were phenotypically characterized by a combination disk test using Neo-Sensitabs™ (Rosco, Denmark), and OXA-48 1.6% (5/312), MBL 0.6% (2/312), and KPC and OXA-48 0.3% (1/312) were identified classes of carbapenemases (Table 3).

### 3.4. Antibiotic Resistance Pattern of *MDRE and CPE*

The majority of isolated MDR *Enterobacteriaceae* showed a resistance level of 92.5% for amoxicillin with clavulanic acid followed by 77.6% for ampicillin and 75.7% for sulfamethoxazole-trimethoprim. Among the isolates, *E. coli* showed the highest resistance to ampicillin (98.0%) followed by amoxicillin with clavulanic acid (93.4%). The second most common isolate K*. pneumoniae* showed 82.4% to amoxicillin with clavulanic acid, 85.3% to sulfamethoxazole-trimethoprim, and 76.5% to ceftriaxone. However, all isolates showed a relatively low level of resistance against amikacin (1.4%), meropenem (3.3%), and imipenem (4.7%) (Table 4).

**Figure 1:**
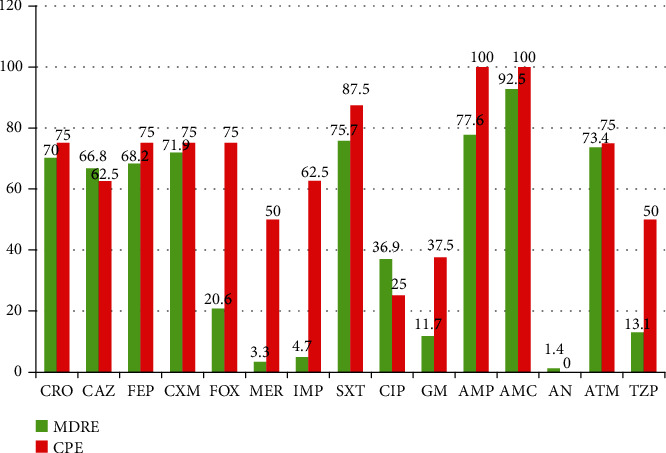
Antimicrobial resistance pattern of MDRE and CPE among clinical specimens at ICL, Addis Ababa, Ethiopia.

The antibiotic resistance rates of CPE isolates were significantly higher than other MDRE including ampicillin (100% versus 77.6%), cefoxitin (75% versus 20.6%), and piperacillin/tazobactam (50% versus 13.1%). As shown in Figure 1, 100% resistance was observed to ampicillin and amoxicillin-clavulanic acid. Moreover, only 25% of strains were resistant to ciprofloxacin, and no resistance was observed to amikacin.

### 3.5. Carbapenemase-Producing *Enterobacteriaceae* and MDR Level among Different Specimens

**Figure 2:**
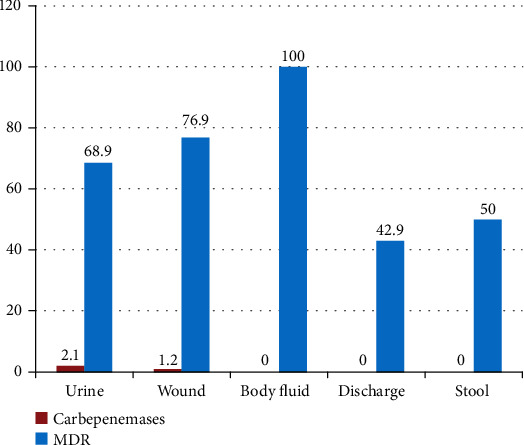
Distribution of carbapenemase-producing *Enterobacteriaceae* and MDR isolates among various clinical specimens at ICL, Addis Ababa, Ethiopia.

Among specimens investigated, MDR-producing *Enterobacteriaceae* were found predominantly in body fluid 100% (10/10) whereas majority of carbapenemase-producing *Enterobacteriaceae* were isolated from urine 2.1% (5/241) (Figure 2). All carbapenemase producing Enterobacteriaceae were MDR.

### 3.6. Association of Independent Variables with MDRE

In multivariable analysis, the odds of having MDRE were 8.82 times (AOR = 8.824, 95% CI: (3.769, 20.654), *P* < 0.001) more likely among inpatients than outpatients. Moreover, the remaining variables such as age, sex, and specimen types do not have statistically significant association with MDRE.

## 4. Discussion

Beta-lactamase-producing *Enterobacteriaceae* have become a global threat. Production of carbapenemase with the emergence of antibacterial resistance is the most important cause of empirical treatment failures. Moreover, current knowledge of the prevalence of MDR Gram-negative bacteria is important to understand their epidemiology and the disease burden and also to strengthen hospital infection control strategy [3].

In this study, MDR (nonsusceptible to ≥1 agent in ≥3 antimicrobial categories) was observed in 68.6%. This finding was in line with studies conducted in Addis Ababa (68.3%) [17], Gondar (68.0%) [18], Debre Markos (72.2%) [19], and Nepal (64.0%) [20] while it was lower than studies done in Ethiopia such as Addis Ababa (94.5%) [21], Gondar (87.4%) [22], and Bahir Dar (93.1%) [23] and studies done in Mozambique (88.2%) [24], Sierra Leone (85.7%) [25], Iran (91.5%) [26], and Nepal (96.8%) [27]. The reason for the variation might be due to differences in the AST method and the presence of beta-lactamase-producing organisms which are resistant to multiple classes of antibiotics. Compared with the present study, MDR isolates were lower in Jimma (59.3%) [28], Italy (62.0%) [29], Nepal (54.2%) [20], and USA (19.1%) [30]. The difference in the magnitude of MDR might be due to the definition used to classify isolates into MDR, patient condition, and presence of carbapenemase-producing isolates in these studies. Repeated, inappropriate, and incorrect use of antimicrobial agents in empirical treatment and poor infection control strategies, in turn, raise the prevalence of resistant bacteria in the community.

The highest MDR strains were detected from *Enterobacter* spp. (90.9%) and *Citrobacter* spp (81.8%) which is comparable to other studies done in Jimma [28] and Nepal [31]. Different studies showed different pathogens as predominant MDR isolates, *K. pneumoniae* and *E. coli* in Gondar [22], Sierra Leone [24], Iran [32], and Nepal [20]. The difference might be due to these bacteria being found in both hospital- and community-acquired infections. In addition, these bacteria are resistant to multiple groups of antimicrobial agents which makes treatment difficult [20].

Although no nationwide study has been conducted so far for the detection of carbapenemase-producing *Enterobacteriaceae* in Ethiopia, some studies have been done in some parts of the country [19, 21, 22].

The present study showed that out of seventeen carbapenemase-suspected *Enterobacteriaceae*, 8 (2.6%) were carbapenemase producers which was in line with a study done in Addis Ababa (2%) [21], Gondar (2.73%) [22], Morocco (2.8%) [33], Taiwan (2.5%) [34], and Jordan (2.8%) [35]. The magnitude of carbapenemase-producing *Enterobacteriaceae* in the current study was lower when compared with the finding in Addis Ababa 12.12% [36], Uganda 22.4% [37], India 23% [38], and Sudan 56% [39]. The difference in these findings might be due to method difference and the patient condition (in which others only include inpatient and isolates resistant to at least two 3GC (third-generation cephalosporin)). Furthermore, the variation might be due to the difference in local antibiotic prescribing habits and infection control program in different health facilities [39].

In this study, OXA-48 enzyme was the most prevalent carbapenemase in Addis Ababa which was supported by a study done by Manenzhe et al. reporting that oxacillinases especially OXA-48 were the most predominant type of carbapenemase in Africa [40]. Studies in Egypt [41] and Spain [42] also showed similar findings. Prevalence and types of carbapenemases can be affected by the difference in phenotypic methods, difference in the study area, and prevalence of carbapenemase genes in different countries [39]. However, surveillance, hand hygiene, and appropriate antibiotic usage are part of an effective approach in reducing the dissemination of these pathogenic organisms [43].

Co-production of KPC and OXA-48 enzymes in the current study was found in one carbapenem-resistant isolate. A comparable result was reported in Uganda [37], but this finding was not in agreement with the finding in Nigeria [44] and Thailand [45] which showed coproduction of NDM and KPC enzyme in one isolate, this is because NDM was the predominant enzyme in these countries. Co-production of two carbapenemase enzymes by one bacterium results in the inactivation of beta-lactamase inhibitors and high-level resistance to the carbapenems as well [42].

The present study showed that the most common carbapenemase-producing *Enterobacteriaceae* were *K. pneumonia* which was agreed with a study done in Addis Ababa [36], Thailand [45], and Jordan [35]. However, it was inconsistent compared to the study done in Gondar [22], Sudan [39], and Pakistan [46] indicating that the principal carbapenemase-producing pathogen was *E. coli* than *K. pneumonia.*

Majority of carbapenemase-producing *Enterobacteriaceae* in the present study was isolated from urine specimens. This agrees with the previous studies conducted in Ethiopia, Sudan, and Kenya [36, 39, 47]. This might be due to the larger number of urine specimens included during the study period and also majority of study participants were females who are at high risk of infections especially urinary tract infection.

In the present study, the overall antibiotic resistance rates of carbapenemase-producing isolates were significantly higher for more than half of tested antibiotics including ampicillin (100%), amoxicillin with clavulanic acid (100%), sulfamethoxazole-trimethoprim (87.5%), and ceftriaxone and cefepime (75.0%). This finding was comparable with a study in Gondar: ampicillin (100%), sulfamethoxazole-trimethoprim (100%), amoxicillin with clavulanic acid (100%), ceftazidime (80%), gentamycin (80%), cefepime (60%), and ceftriaxone (60%) [22]; Jordan [35]; and Bangladesh [48] showing all carbapenemase-producing isolates indicating the highest resistance to amoxicillin with clavulanic acid (100%), ampicillin (100%), and cefepime (100%). These findings indicated that carbapenemase-producing *Enterobacteriaceae* were the major cause of resistance to various antibiotic classes.

This study also showed that carbapenemase-producing *Enterobacteriaceae* were 100% sensitive to amikacin and 75.0% to ciprofloxacin. This was fairly similar with a study conducted in Nigeria: ciprofloxacin (57.0%) [49], Tanzania: ciprofloxacin (66.5%) [50], and Nepal: amikacin (91.8%) [20]. Antibiotic treatment options for carbapenem-resistant bacteria are limited and are highly costly. However, combination therapy with active drugs such as colistin, tigecycline, and fluoroquinolones can be alternative antibiotics [51].

## 5. Conclusion and Recommendation

In this study, a relatively higher prevalence of MDRE and significant prevalence of CPE were observed, and the highest resistance was recorded against ampicillin, amoxicillin with clavulanic acid, and sulfamethoxazole-trimethoprim. On the other hand, the better treatment option for CPE is amikacin and ciprofloxacin. The phenotypic confirmatory test indicated that more oxacillinase-48- (OXA-48-) producing *Enterobacteriaceae* was detected in this study. Detection of carbapenemase-producing *Enterobacteriaceae* is important for implementing appropriate antimicrobial therapy and in controlling the spread of the infection. Furthermore, continuous screening and investigations, including genotypic characterization of carbapenemase-producing *Enterobacteriaceae*, are required for the prevention and control of the spread of antimicrobial-resistant pathogens.

## 6. Limitation of the Study

- Molecular technique was not used for the characterization of carbapenemase-producing*Enterobacteriaceae* due to financial constraints.

## Figures and Tables

**Table 1:** Distribution of Enterobacteriaceae isolate among various specimen types at ICL, Addis Ababa, Ethiopia.

	Urine	Pus	Body fluid	Ear discharge	Eye discharge	Stool	Total
*E. coli*	192 (84.9)	25 (11.1)	7 (3.1)	1 (0.4)	2 (0.9)	0 (0.0)	226
*K. pneumoniae*	26 (60.5)	12 (27.9)	2 (4.7)	1 (2.3)	1 (2.3)	0 (0.0)	43
*Enterobacter* spp.	5 (45.5)	6 (54.5)	0 (0.0)	0 (0.0)	0 (0.0)	0 (0.0)	11
*Citrobacter* spp.	9 (81.8)	2 (18.2)	0 (0.0)	0 (0.0)	0 (0.0)	0 (0.0)	11
*P. mirabilis*	2 (33.3)	2 (33.3)	0 (0.0)	2 (33.3)	0 (0.0)	0 (0.0)	6
*Shigella* spp.	0 (0.0)	0 (0.0)	0 (0.0)	0 (0.0)	0 (0.0)	2 (100)	2
Other isolates	7 (53.8)	5 (38.5)	1 (7.7)	0 (0.0)	0 (0.0)	0 (0.0)	13
Total	241	52	10	4	3	2	312

Note: ^∗^other isolates are *Salmonella spp*, *Providencia spps*, *M. morganii*, and *Serratia* spp.

**Table 2:** Multidrug resistance pattern of *Enterobacteriaceae* at ICL, Addis Ababa, Ethiopia.

Isolates (*n*)	Level of antibiotic resistance (*n* (%))									
	R0	R1	R2	R3	R4	R5	R6	R7	≥R8	Total MDR isolates (≥3)
*E. coli* (226)	30 (13.3)	20 (8.8)	25 (11.1)	28 (12.4)	12 (5.3)	29 (13.7)	33 (14.6)	28 (12.4)	21 (9.3)	151 (66.8)
*K. pneumoniae* (43)	3 (0.0)	4 (0.0)	2 (0.0)	6 (13.9)	2 (4.6)	2 (4.6)	9 (20.9)	8 (18.6)	7 (16.3)	34 (79.1)
*Enterobacter* spp. (11)	0 (0.0)	0 (0.0)	1 (9.1)	0 (0.0)	2 (18.2)	0 (0.0)	1 (9.1)	3 (27.3)	4 (36.4)	10 (90.9)
*Citrobacter* spp. (11)	1 (9.1)	0 (0.0)	1 (9.1)	2 (18.2)	2 (18.2)	1 (9.1)	4 (36.4)	0 (0.0)	0 (0.0)	9 (81.8)
*P. mirabilis* (6)	2 (33.3)	0 (0.0)	1 (16.7)	0 (0.0)	2 (33.3)	0 (0.0)	1 (16.7)	0 (0.0)	0 (0.0)	3 (50.0)
*Shigella* spp. (2)	1 (50.0)	0 (0.0)	0 (0.0)	0 (0.0)	0 (0.0)	1 (50.0)	0 (0.0)	0 (0.0)	0 (0.0)	1 (50.0)
*M. morganii* (5)	2 (40.0)	1 (20.0)	0 (0.0)	1 (20.0)	1 (20.0)	0 (0.0)	0 (0.0)	0 (0.0)	0 (0.0)	2 (40.0)
*Providencia* spp. (5)	0 (0.0)	2 (40.0)	0 (0.0)	1 (20.0)	1 (20.0)	1 (20.0)	0 (0.0)	0 (0.0)	0 (0.0)	3 (60.0)
*Serratia* spp. (3)	0 (0.0)	2 (66.6)	0 (0.0)	1 (33.3)	0 (0.0)	0 (0.0)	0 (0.0)	0 (0.0)	0 (0.0)	1 (33.3)
Total (*n* = 312)	39 (12.5)	29 (9.3)	30 (9.6)	39 (12.5)	22 (7.1)	34 (10.9)	48 (15.4)	39 (12.5)	32 (10.3)	214 (68.6)

Note: R0: resistance to no antibiotics; R1-8: resistance to 1, 2, 3, 4, 5, 6, 7, and 8 antibiotics; ≥R3: resistance to 3 or more antibiotics from different classes.

**Table 3:** Distribution of carbapenemase-producing *Enterobacteriaceae* at ICL, Addis Ababa, Ethiopia.

Isolates (number)	mCIM	OXA-48	MBL	KPC+OXA-48
*E. coli* (226)	3 (1.3)	3 (1.3)	0 (0.0)	0 (0.0)
*K. pneumoniae* (43)	4 (9.8)	2 (4.9)	1 (2.4)	1 (2.4)
*Enterobacter Spp*. (11)	1 (9.1)	0 (0.0)	1 (9.1)	0 (0.0)
*Citrobacter Spp*. (11)	0 (0.0)	0 (0.0)	0 (0.0)	0 (0.0)
*P. mirabilis* (6)	0 (0.0)	0 (0.0)	0 (0.0)	0 (0.0)
*Shigella* Spp. (2)	0 (0.0)	0 (0.0)	0 (0.0)	0 (0.0)
Other Spp. (13)	0 (0.0)	0 (0.0)	0 (0.0)	0 (0.0)
Total (*n* = 312)	8 (2.4)	5 (1.5)	2 (0.6)	1 (0.3)

**Table 4:** Antibiotic resistance pattern of MDRE at ICL, Addis Ababa, Ethiopia.

Isolates (*N*)	CRO	CAZ	FEP	CXM	FOX	MER	IMP	ETP	SXT	CIP	GM	AMP	AMC	AN	ATM	TZP	Total (*n*)
*E. coli*	110 (72.8)	108 (71.5)	110 (72.8)	107 (70.9)	20 (13.2)	2 (1.3)	1 (0.7)	5 (3.3)	114 (75.5)	56 (37.1)	15 (9.9)	148 (98.0)	143 (93.4)	1 (0.7)	113 (74.8)	15 (9.9)	151
*K. pneumoniae*	26 (76.5)	25 (73.5)	25 (73.5)	27 (79.4)	7 (20.6)	4 (11.8)	5 (14.7)	4 (11.8)	29 (85.3)	14 (41.2)	5 (14.7)	NA	28 (82.4)	0 (0.0)	26 (76.5)	8 (23.5)	34
*Enterobacter Spp.*	8 (80.0)	5 (50.0)	7 (70.0)	8 (80.0)	9 (90.0)	0 (0.0)	0 (0.0)	1 (10.0)	8 (80.0)	3 (30.0)	3 (30.0)	10 (100.0)	10 (100)	0 (0.0)	8 (80.0)	2 (20.0)	10
*Citrobacter* spps.	2 (22.2)	2 (22.2)	1 (11.1)	4 (44.4)	4 (44.4)	0 (0.0)	2 (22.2)	0 (0.0)	5 (55.6)	2 (22.2)	0 (0.0)	NA	9 (100.0)	0 (0.0)	5 (55.6)	1 (11.1)	9
*P. mirabilis*	1 (33.3)	0 (0.0)	1 (33.3)	3 (100)	1 (33.3)	0 (0.0)	0 (0.0)	0 (0.0)	1 (33.3)	1 (33.3)	0 (0.0)	2 (66.7)	2 (66.7)	0 (0.0)	1 (33.3)	0 (0.0)	3
*Other*	4 (57.1)	3 (42.9)	3 (42.9)	5 (71.4)	3 (42.9)	1 (14.9)	2 (28.6)	2 (28.6)	5 (71.4)	3 (42.9)	2 (28.6)	6 (85.7)	6 (85.7)	2 (28.6)	4 (57.1)	2 (28.6)	7
Total resistance	150 (70.0)	143 (66.8)	146 (68.2)	154 (71.9)	44 (20.6)	7 (3.3)	10 (4.7)	12 (5.6)	162 (75.7)	79 (36.9)	25 (11.7)	166 (77.6)	198 (92.5)	3 (1.4)	157 (73.4)	28 (13.1)	214

Note: CRO: ceftriaxone; CAZ: ceftazidime; FEP: cefepime; CXM: cefuroxime; FOX: cefoxitin; MEM: meropenem; IMP: imipenem; SXT: trimethoprim-sulfamethoxazole; CIP: ciprofloxacin; GM: gentamicin; AMP: ampicillin; AMC: amoxicillin-clavulanic acid; AN: amikacin; ATM: aztreonam; TZP: piperacillin/tazobactam; ETP: ertapenem.

## Data Availability

Data supporting the conclusion of this article are within the manuscript.

## Abbreviations

AST: Antibiotic susceptibility testing
ATCC: American Type Culture Collection
CLSI: Clinical and Laboratory Standards Institute
CRE: Carbapenem-resistant *Enterobacteriaceae*
CPE: Carbapenemase-producing *Enterobacteriaceae*
ESBL: Extended spectrum beta-lactamase
EUCAST: European Committee on Antimicrobial Susceptibility Testing
ICL: International Clinical Laboratories
IMP: Imipenemase
KPC: Klebsiella pneumoniae carbapenemase
MDR: Multidrug resistance
MIC: Minimum inhibitory concentration
MBL: Metallo beta-lactamase
mCIM: Modified carbapenem inactivation method
OXA-48: Oxacillinase-48 carbapenemase
VIM: Veronica integron metallo-*β*-lactamase types.
